# The ethical challenges of the clinical introduction of mitochondrial replacement techniques

**DOI:** 10.1007/s11019-015-9656-3

**Published:** 2015-08-04

**Authors:** John B. Appleby

**Affiliations:** Centre of Medical Law and Ethics in the Dickson Poon School of Law, King’s College London, London, UK

**Keywords:** Donation, Embryo, Genetics, IVF, Three-parent

## Abstract

Mitochondrial DNA (mtDNA) diseases are a group of neuromuscular diseases that often cause suffering and premature death. New mitochondrial replacement techniques (MRTs) may offer women with mtDNA diseases the opportunity to have healthy offspring to whom they are genetically related. MRTs will likely be ready to license for clinical use in the near future and a discussion of the ethics of the clinical introduction of MRTs is needed. This paper begins by evaluating three concerns about the safety of MRTs for clinical use on humans: (1) Is it ethical to use MRTs if safe alternatives exist? (2) Would persons with three genetic contributors be at risk of suffering? and (3) Can society trust that MRTs will be made available for humans only once adequate safety testing has taken place, and that MRTs will only be licensed for clinical use in a way that minimises risks? It is then argued that the ethics debate about MRTs should be reoriented towards recommending ways to reduce the possible risks of MRT use on humans. Two recommendations are made: (1) licensed clinical access to MRTs should only be granted to prospective parents if they intend to tell their children about their MRT conception by adulthood; and (2) sex selection should be used in conjunction with the clinical use of MRTs, in order to reduce transgenerational health risks.

It is estimated that about 1 in every 6500 children in the UK is affected by a mitochondrial DNA (mtDNA) disease, which makes this group of neuromuscular diseases one of the most prevalent (Schaefer et al. 2008; DH 2014a). These diseases often result in considerable suffering and even death. However, the development of new mitochondrial replacement techniques (MRTs) could allow parents to conceive children to whom they are genetically related and who will not suffer from these diseases. The two most promising MRTs in development are maternal spindle transfer (MST) and pronuclear transfer (PNT), and no children have been born with either of these techniques to date.

Following consultations by the Nuffield Council on Bioethics (NCB) (2012), the UK Human Fertilisation and Embryology Authority (HFEA) (2013a) and the UK Department of Health (2014a, b), the UK parliament voted in early 2015 to approve regulations that permit the licensed clinical use of MST and PNT (HFEA 2015). These new MRT regulations entitled *The Human Fertilisation and Embryology (Mitochondrial Donation) Regulations 2015* (HFE Regulations 2015), come into force on 29 October 2015 and make amendments to the Human Fertilisation and Embryology Act 1990 (HFE Act 1990) (as amended). However, before MRTs can transition from lab to clinic in the UK, the HFEA must first develop a licensing framework to evaluate applications from clinicians wishing to use these techniques, on a case by case basis (HFEA 2015). This framework must be able to account for the many ethical and scientific challenges that accompany the use of these techniques and according to the HFEA must ‘ensure that any children born have the best chance of a healthy life’ (HFEA 2015). Policymakers and research institutes from the UK, Sweden, and the USA, among others, have recommended that careful ethical consideration should be given when determining the conditions under which MRTs should first be made available in clinics, in order to minimise human exposure to risks wherever possible (NCB 2012: pp. 1–14; SNCME 2013; FDA 2014a, b, c).

A small but valuable body of literature discussing the ethics of using MRTs on humans currently exists. However, despite the UK Parliament’s recent approval of the HFE Regulations 2015, new research on this area of ethical debate remains scarce. This paper makes an important contribution to the existing literature by providing an up-to-date and critical discussion of several of the key ethical concerns (especially the safety and risks) surrounding the clinical introduction of MRTs. In response to the HFEA’s movement toward licensing MRT’s for clinical use, this paper also recommends ethically important licensing conditions, which are aimed at helping the HFEA (and potentially other international regulators) reduce the possible risks that accompany the use of these techniques on humans.

After providing a brief background and scientific overview for the current ethical debate surrounding MRTs, this paper begins by evaluating three central concerns about the safety of allowing MRTs to be used in clinics: (1) Is it ethical to use MRTs if other safe alternatives exist? (2) Would persons with three genetic contributors be put at risk of psychosocial or physical suffering? and (3) Can society trust that MRTs will be made available for human use only once adequate scientific research has taken place, and that the HFEA’s future licensing framework for clinics will be robust enough to minimise the risks to future persons born by these techniques? After responding to these concerns, it is argued that the ethics debate should now be re-oriented towards recommending ways that regulators and clinicians can reduce or eliminate the possible health risks of the first clinical use of MRTs, as this is an area in urgent need of further attention. One way to reduce the possible health risks and make the prospective use of MRTs more ethical, is to place conditions on the circumstances under which clinicians are granted licenses for the clinical use of MRTs. In response to the HFEA’s need to develop a new licensing framework for the safe and ethical clinical use of MRTs, two key recommended licensing conditions are put forward.

The first is that MRTs should only be licensed for clinical use on the condition that prospective parents are only granted clinical access to these techniques if they intend to tell their future MRT-conceived1^1^ children about the method of their conception by adulthood. The advantages of such a policy would be that MRT-conceived persons would have improved autonomy to care for themselves and to make more informed reproductive choices in life. In addition, long-term medical follow-up and social science research on MRT-conceived persons is important (Barber and Border 2015; HFEA 2013a) (but not required) (DH 2014b: p. 42; HFE Regulations 2015) and it would be unethical to monitor or conduct any research on these persons once they are adults, if they are not first adequately informed about this.^2^

The second condition is that licenses should only be granted to clinics if sex selection will be used to increase the likelihood^3^ that only male embryos will be created and implanted in the first clinical use of MRTs. Because mitochondria are maternally inherited, any male offspring created would be at no risk of passing on any inheritable health complications associated with their mitochondria which may have been caused by the use of MRTs. Sex selection should play an important role in minimising the transgenerational health risks of any clinical use of MRTs, at least until more is known about their transgenerational safety. Much of this paper focuses on UK policy in relation to MRTs; however the general discussion is relevant to international policy, in both clinical and research settings.

## Background and scientific overview

### How mtDNA diseases occur

Mitochondria are cellular organelles which generate energy for cellular functions. Every mitochondrion carries many copies of its mtDNA and mutations in the mtDNA are either created spontaneously during mtDNA replication or they are maternally inherited. Mutations can be present in our mtDNA in one of two ways: (1) the mutations exist in 100 % of the mtDNA in our bodies, which is known as homoplasmy; or, (2) our bodies carry a mixture of healthy and mutant mtDNA, which is known as heteroplasmy. Intending mothers with a homoplasmic mtDNA mutation (e.g. Leber hereditary optic neuropathy, or LHON) will always pass on the mutation to their offspring (McFarland et al. 2002: pp. 145–6);^4^ however, depending on the mutation, offspring may or may not suffer medical problems. Determining whether or not someone is likely to pass on a heteroplasmic mtDNA disorder to their children is also not always straightforward. Heteroplasmic mtDNA mutations (e.g. neurogenic muscle weakness, ataxia, retinis pigmentosa, or NARP) are among the most common and while these mutation(s) will always be inherited by offspring, the mutation loads^5^ that are present in those offspring will often vary from person to person and depend on their age. The variability of mutant loads among heteroplasmic mtDNA mutant carriers is the result of mosaicism in the developing embryo, which is caused by genetic bottlenecks during mitochondrial division.^6^ The seriousness of an mtDNA disorder typically correlates with the mutant load and the mutation itself. As a result, a considerable amount of uncertainty often exists around the question of whether or not mothers’ mtDNA mutation(s) will manifest in a harmful way when inherited by their offspring (Bredenoord et al. 2008a; NCB 2012: p. 26).

### Maternal spindle transfer (MST) and pronuclear transfer (PNT)

It is important to clarify the basic differences between MST and PNT. The technique of MST involves removing the maternal spindle (the nuclear DNA) of an intending mother’s egg with diseased mitochondria, and transferring that maternal spindle into an enucleated donor egg with healthy mitochondria. The reconstructed egg is then fertilised with the intending father’s sperm to create a healthy embryo which can later be transferred into the womb. In contrast, PNT involves removing the pronuclei (the nuclear DNA from the intending mother’s egg and intending father’s sperm) from an embryo carrying diseased mitochondria and transferring those pronuclei into an enucleated embryo (created using a healthy donor egg and the intending father’s sperm) carrying healthy mitochondria. (Bredenoord et al. 2010: p. 1354).^7^ The reconstructed embryo can later be transferred into the womb.

As mentioned in the introduction, no children have been born via MST or PNT and the full extent of risks associated with their use is not known (NCB 2012; Baylis 2013; HFEA 2013a; Knoepfler 2014). Since 2011 the HFEA has annually published a ‘Review of scientific methods to avoid mitochondrial disease’ which considers developments in the safety and efficacy of MRTs. In 2011 the HFEA reported that a panel of scientific experts concluded that the ‘evidence currently available does not suggest that the techniques are unsafe’ (HFEA 2014, p 29). Some have questioned whether it is ethical to discuss the use of techniques, such as MRTs, until enough evidence is available to say that they are safe, (Morrow 2014a) and have argued that the public should not be satisfied with the rigour of the HFEA’s research on safety to date (Taylor 2015). Others have pointed out that until a novel medical technique has been used on a human for the first time, it almost impossible to be certain of that technique’s risks and safety for human use (as discussed in NCB 2012: p. xv). Nevertheless, the HFEA has made clear that it will continue to monitor ongoing and future research developments that bear on the safety and efficacy of both techniques and that no licenses for the clinical use of MRTs will be granted until scientific experts ‘advise the HFEA that these [research] results are reassuring’ (HFEA 2013a; p. 29).

Serious safety concerns about medical techniques involving mitochondrial transfer were initially raised in the US in 2001 (NCB 2012). Between 1997 and 2001, approximately 30 babies were born worldwide (but primarily in the US) following the use of an in vitro procedure known as cytoplasmic transfer (CT), which is a similar but different technique to MST and PNT (NCB 2012). CT entails taking cytoplasm containing healthy mitochondria from a donor’s egg and transferring this cytoplasm into a recipient’s egg; thus, creating an egg that carries mitochondria from both the intending mother and the donor. This technique was developed as a way of improving the fertility of the eggs of women who suffer from infertility problems associated with repeatedly unsuccessful embryo implantation or inadequate embryo development (NCB 2012). The science behind CT as a treatment is still not entirely understood; however, by transferring healthy mitochondria into an egg carrying unhealthy mitochondria, CT has been described as a way of potentially reducing the detrimental effects of mitochondrial disease transmission (NCB 2012). The US Food and Drug Administration (FDA) banned the use of CT in 2001 (NCB 2012) following reports that one of the fifteen children born from a US clinic was diagnosed with pervasive developmental disorder (PDD; Barritt et al. 2000, 2001), and two subsequent CT pregnancies from the same clinic were found to be affected with Turner syndrome (one pregnancy was terminated and the other resulted in a miscarriage) (Barritt et al. 2000, 2001; NCB 2012).^8^ As a consequence, CT fell into scientific disrepute (NCB 2012). There appears to be no published follow-up research on the US children born following CT and there is no central register that is kept as a record that these children were born this way.

In February 2014 the FDA held meetings to discuss new developments in the field of MRTs (e.g. MST and PNT) and how future research could be undertaken in a safe and ethical way (FDA 2014a, b). The FDA Advisory Committee found that more scientific research with MRTs was needed, both on animals and in vitro using human embryos, before these techniques could be used in a human trial (FDA 2014b). Therefore, the FDA’s advisory committee and the HFEA appear to currently share the view that more research is needed before MRTs are used on humans and both have an ongoing scientific review process in place. The Institute of Medicine (IOM), an independent US non-profit organisation, has recently begun a study commissioned by the FDA to ‘produce a consensus report regarding the ethical and social policy issues related to genetic modification of eggs and zygotes to prevent transmission of mitochondrial diseases’ (IOM 2015). The first meetings were held in January 2015 and the IOM committee that is leading the study has indicated that its report will be ready later in 2015.

In contrast to the US, the UK has had a long-running ethical debate surrounding the prospect of using MST and PNT in clinics. In autumn 2011 the NCB launched a consultation on ‘Novel techniques for the prevention of mtDNA disorders: an ethical review’, which was published in May 2012. In early 2012 the HFEA also launched a public consultation entitled ‘Medical frontiers: debating mitochondrial replacement’ and the findings were published in March 2013 in a report entitled ‘Mitochondria replacement consultation: advice to government’. The NCB and HFEA reports expressed support for further research into the safety and efficacy of MRTs, and the HFEA report also advised the government that there is general support for permitting MRTs in the UK. The UK government then drafted regulations to permit the licensed clinical use of MRTs and the Department of Health held a public consultation on these draft regulations in early 2014. Based on the consultation’s findings the Department of Health decided to put the draft regulations before Parliament in early 2015 and by February 24th both Houses of Parliament had approved them. As noted earlier, the HFE Regulations 2015 are set to come into force in the UK on 29 October 2015 and the HFEA must develop a robust licensing framework that can review applications from clinicians wishing to use these techniques.

## Ethical challenges to the clinical use of MRTs

The ethical debate surrounding the clinical introduction of MRTs has generally been centred on three concerns outlined earlier about the safety of these techniques. This section aims to clarify whether or not these concerns are well-founded and worthy of attention.

### Alternatives to MRTs

It has been argued that MRTs are not an ethical means of reproduction so long as questions remain about their safety and other safe means of reproduction already exist (Baylis 2013: pp. 532–3).^9^ For example, the use of donor eggs and embryos to conceive children is known to be safe following years of clinical use. In contrast, PNT and MST have not been used on humans to prove their safety. Until MRTs are first used on humans to demonstrate their safety, the use of donor eggs and embryos will continue to be viewed by many as a safer way of having children. A report by the NCB (2012: p. 67) discusses how some have objected that even using MRTs on a small number of humans would be unethical. This objection is based on the view that it will *always* be unethical to create a human with MRTs so long as risks exist and other safe alternatives are available. However, this objection raises an important question: can safe alternatives (e.g. using donated eggs or embryos) offer the same reproductive benefits that some prospective parents want and that MRTs promise?

Unlike the use of donor eggs or embryos, MRTs provide a genetic link between mothers and their children. Some may feel that it is important to have a genetic link with their future child and that having this genetic link outweighs most disadvantages (e.g. health risks and high financial cost) associated with MRTs; thus, for these intending mothers using egg or embryo donation is not a suitable alternative. Therefore, what other safe alternatives could allow an intending mother to have a child to whom they are genetically linked?

Pre-implantation genetic diagnosis (PGD) and prenatal diagnosis (PND) are two techniques that can sometimes be used as alternatives to MRTs, in order to help intending mothers have healthy children to whom they are genetically related. The process of PGD involves removing a cell from an embryo in order to test that embryo for the presence of harmful concentrations of mtDNA mutations that could result in a mtDNA disease. This procedure is conducted prior to the embryo being transferred into the uterus and can be used to select an embryo that is considered to be the least likely to result in a person that will suffer from a mtDNA disease. Similarly, PND is used to test the cells of a fetus to determine if it is likely to have a mtDNA disease based on the concentration of mutant mtDNA that is present in its cells during prenatal development. Prospective mothers can then abort the pregnancy if they do not want to give birth to a child who is likely to have a mtDNA disease.

However, the predictive uncertainty associated with these techniques means that they are not always reliable methods of avoiding the creation of children who will suffer from mtDNA diseases (Bredenoord et al. 2008a). PGD and PND are of no use for intending mothers with homoplasmic mtDNA diseases because their eggs and embryos will always have a 100 % mutation load of diseased mtDNA (Braude and Lovell-Badge 2014). For intending mothers with heteroplasmic mutations, PGD may also reveal that it is impossible for them to produce eggs with safe levels of mutant mtDNA. Finally, any female offspring born following PGD or PND may also be liable to pass on mtDNA diseases to their offspring, due to the ongoing presence of some inherited mutant mtDNA in their bodies (Bredenoord et al. 2008b).

Therefore, in some cases, PGD and PND can help intending mothers have children to whom they are genetically related and who will not suffer from mtDNA diseases. For other intending mothers PGD and PND will not be viable alternatives to help them have healthy children. This latter group of intending parents will be left with the remaining options of either not reproducing or waiting for MRTs to become clinically available. In other words, MRTs may be the only chance for some intending mothers to have children to whom they are genetically related. Evidence suggests that rather than abstain from reproducing, some parents would be willing to use MRTs if they become clinically available (NCB 2012: p. 61). However, some (Baylis 2013; Taylor 2015) argue that existing safety concerns about MRTs makes them an unethical reproductive option for anyone to choose (even if this means not having children). Therefore, what are these safety concerns and how serious are they?

### MRT safety and offspring with three genetic contributors

One of the most common safety concerns associated with MRTs is that they will create persons with three genetic ‘parents’ and that having three genetic ‘parents’ could cause a person to suffer (NCB 2012: pp. 32–6; Baylis 2013: p. 522; Donnelly 2014; Gallagher 2014).^10^ However, this claim requires clarification. There is no reason that any particular parenting arrangement will follow from the use of MRTs (Johnson 2013) and it is misleading to suggest that children conceived by MRTs will have three *parents*. The wording of ‘three genetic parents’, has often been used by the media and researchers to describe how MRT-conceived children will have three genetic contributors (Donnelly 2014; Caldwell 2015). It is this link between the use of MRTs and the creation of children with genetic contributions from three individuals that has caused ethical tensions to arise. Two predominant safety concerns exist about MRT-conceived children having three genetic contributors.

The first concern is that MRT-conceived children could experience some form of *psychosocial* suffering as a result of having genetic ties to three persons (as discussed in NCB 2012: p. 71).^11^ For example, one worry is that children might have a troubled relationship with their parents or struggle to develop their identity, as a result of knowing they share a mitochondrial genome with a donor. Currently no empirical evidence exists about the psychosocial wellbeing of MRT-conceived children, which makes it difficult to determine if any of the above risks will materialise. However, empirical evidence does exist from social science studies on children conceived via gamete donation. This psychosocial evidence does not indicate that gamete donor-conceived children experience problems with their identity development, but it does show that donor-conceived children have functional relationships with their parents (Ilioi and Golombok 2014; Shelton et al. 2009). Evidence from families created by gamete donation can provide valuable insight into the psychosocial development of children who share genetic ties with others who do not necessarily occupy parental roles in their lives.^12^ When discussing the prospect of creating children using MRTs, the existing evidence about children conceived via gamete donation should help to allay any concerns about how vulnerable these prospective children might be to suffering from psychosocial problems; thus, at this time it would be unreasonable to prohibit the use of MRTs, on the grounds of contestable safety claims such as these.

The second concern is that the *physical* wellbeing of MRT-conceived children could suffer because they have three genetic contributors.^13^ For instance, one possible risk is that a donor’s mtDNA could potentially fail to function properly with the nuclear genes contributed by the intending parents (HFEA 2013b; Knoepfler 2014; Morrow 2014b).^14^ If donor mtDNA did prove to be incompatible with the nuclear DNA of the intending parents, the resulting child might suffer from health complications. Another concern is that during PNT or MST, some of the intending mother’s diseased mitochondria could be inadvertently transferred into the ‘healthy’ embryo or egg, respectively. While the presence of a very small amount of diseased mtDNA may not be a health risk for the carrier, it could pose a health risk (i.e. an mtDNA disease) for that carrier’s offspring. Therefore, it appears that MRT-conceived children could be exposed to some risks to their physical wellbeing. In light of these possible risks, how, if at all, can the clinical introduction of MRTs proceed in an ethical manner?

### The importance of pre-clinical safety research and risk reduction in clinical settings

With the above health risks in mind, there are two key responsibilities that must be satisfied in order to help make the clinical introduction of MRTs as ethical as possible. The first is to ensure that a rigorous schedule of pre-clinical safety testing takes place for MRTs before they are permitted for human use. The second is to make sure that any clinical use of MRTs includes adequate safeguards to minimise the health risks to future persons wherever possible. However, have both of these responsibilities been adequately accounted for in discussions about MRT research, policy and regulation so far?

The first responsibility seems straightforward; however, some distrust exists towards research on assisted reproductive technologies (ARTs) because there is a long history of some ARTs being introduced into medical practice without having gone through a rigorous schedule of testing or a clinical trial (Dondorp and de Wert 2011; Harper et al. 2011). For example, as recently as 1990, the first child was born after being conceived via intra-cytoplasmic sperm injection (ICSI), without much experimental research being conducted beforehand (Harper et al. 2011: p. 3). In contrast, MRT research has been subject to considerable HFEA oversight, scientific scrutiny and public debate. A schedule of research milestones has been set out by regulators and these milestones must be met in order to ensure the efficacy and safety of MRTs, before they can be licensed for use in a clinical setting (HFEA 2013a: p. 13).

Nevertheless, some critics have argued that the HFEA review process (including the consultation) was methodologically flawed, that it misrepresented public opinion and that it failed to include key research requirements and evidence (Taylor 2015). The HFEA’s scientific review is ongoing, and therefore it is difficult to say with certainty that any particular research or evidence has been definitively discounted from future consideration at this point in time. The HFEA’s consultation also had an independent oversight group to ensure that the process of the consultation was balanced. Furthermore, an independent review of the HFEA’s consultation on MRTs found that some of the report’s methodologies and reporting could be improved, but that overall the process and the final report was sound (Watermeyer and Rowe 2013). Therefore, while some distrust towards ARTs lingers in society, others trust that the safety of MRTs will be thoroughly researched prior to being approved for licensed clinical use on humans.^15^ Importantly, the HFEA must continue working to cultivate and foster the trust of both its sceptics and supporters while it continues to review how MRTs can be licensed for clinical use in the future.

While preparations have been made by the HFEA to fulfil the first responsibility mentioned above, there appears to have only been a limited discussion, so far, about how to satisfy the second one. It is therefore essential that the ethical debate about MRT safety is re-oriented to prioritise and foster discussions about which precautionary measures should be put in place when designing a framework for licensing these techniques, in order to minimise health risks. Next, this paper identifies two recommended conditions that would help to minimise possible mitochondria-related health risks (i.e. those mentioned in previous sections).

### The importance of ‘disclosure’ to persons created via MRTs

The first recommended condition is that prospective parents should only be granted licensed access to MRTs in a clinic if they intend to tell their children, or ‘disclose’,^16^ about their origins (i.e. being MRT-conceived). One benefit of disclosure is that it would save some children from the stress and anxiety of worrying about suffering from the same mtDNA disorders as their mothers.^17^ However, some prospective parents may not wish to disclose to their future MRT-conceived children, as this is also the case with some parents who have children conceived via gamete donation (Readings et al. 2011; Blake et al. 2010). Some prospective parents may prefer non-disclosure because they feel that their use of MRTs is a matter of personal privacy, which they feel their children have no claim to know about. Others who prefer non-disclosure may simply tell their children that they were conceived using IVF, but leave out the fact that an MRT was used. Research has shown that some parents with children conceived via gamete donation have employed similar strategies for non-disclosure and it is possible that some parents with MRT-conceived children will prefer to do the same (Daniels 1997; Freeman 2015; NCB 2013; Readings et al. 2011).

It is important that the first generation of persons conceived via MRTs are disclosed to. Disclosure is important for at least two reasons: (1) the MRT-conceived person’s own medical welfare; and (2) knowledge of having been MRT-conceived enables persons to report any medical problems back to clinicians and researchers for the sake of the wellbeing of future generations who might be conceived via MRTs. Clinicians should only be licensed to treat prospective parents with MRTs if they intend to disclose to their children. Therefore parents’ intention to voluntarily disclose to their future MRT-conceived children should constitute one of the conditions for licensing a clinic to use MRTs.

Disclosure to the first generation of persons conceived via the initial clinical use of MRTs is an important part of safeguarding them from possible MRT-related health risks. For example, the HFEA (2013a: p. 26) also supports the recommendation that future MRT-conceived persons be disclosed to at an early age. Excluding prospective parents from accessing MRTs on the grounds that they do not intend to disclose is justifiable on the basis that: (1) initial access to MRTs will most likely be a limited resource^18^; and (2) it is permissible for the HFEA and clinicians to take a welfare-maximising approach to managing the public health of this research population, which involves only selecting prospective parents who will likely have children who the clinicians believe will fare the best. Knowing one was conceived with an MRT is important when it comes to having an autonomous capacity to care for one’s own wellbeing, especially as an adult. For example, it is important that someone knows that they were conceived with an MRT so that they can inform others (e.g. medical practitioners) who might be caring for their health (e.g. if they are suffering from a health complication related to their mitochondria). Allowing prospective parents to access MRTs, who are not intending to disclose, would increase the possibility that some MRT-conceived children could be deprived of knowing an important part of their medical history. Trying to ensure that future MRT-conceived persons are disclosed to is essential to improving the ethical use of these techniques, as this disclosure will further enable persons to properly care for their own health over the course of their lives.

In addition, the safety of future generations conceived via MRTs is dependent on disclosure to the first generation. To gain a robust understanding of the safety of MRTs, clinicians need to be able to gather information about the health outcomes of the first generation conceived with MRTs, for as long as possible. For example, the UK Department of Health has recently stated that enabling MRT-conceived persons to be followed-up “…is vital if the impact of the mitochondrial donation techniques is to be fully understood” (although, follow-up research will not be required) (DH 2014b: p. 42). In order for clinicians to be able to potentially continue gathering information about the health outcomes of MRT-conceived persons as adults, this cohort will need to have been disclosed to. Otherwise they will never understand the nature of the medical monitoring that may have been part of their lives so far,^19^ and will not realise how they can help future generations by remaining engaged with clinicians.

However, the complexities of disclosure in families should not be understated. Disclosure is typically a process of explanation and a topic of ongoing dialogue between parents and children, and there is usually no clear end date (Blake et al. 2010; Readings et al. 2011; Freeman 2015). Disclosure is not typically a one-off event and the information shared during the initial disclosure may not be something the children can understand the meaning of until they have reached a stage (e.g. adolescence) of adequate psychological development (Blake et al. 2010; Readings et al. 2011; Freeman 2015).^20^ Given the complexities of disclosure in families, it will be important that clinics and the HFEA ensure that parents have access to relevant counselling and resources (e.g. disclosure aids in the form of children’s books).

### Possible objections to the recommendation to use parental intent to disclose as a licensing condition

The first objection to only allowing clinical access to MRTs for parents who intend to disclose might be the following: in the UK prospective parents are not denied access to donor gametes if they do not intend to disclose to their future donor-conceived children, so why should prospective parents accessing MRTs in a clinic be treated any differently? The answer is that because this will be the first clinical use of MRTs, clinicians may be unsure about whether or not the resulting child could still suffer from an inherited mtDNA disorder or some other MRT-related health complication. Therefore, regulators and clinicians are ethically bound to ensure that the limited number of children created via the first use of MRTs have the best chance of being disclosed to, so that they can be aware of these possible health risks. In contrast, robust medical evidence exists regarding the wellbeing of donor-conceived children; thus, there is less medical motivation for disclosure in these cases and they are not comparable to cases of MRT conception in this respect.^21^

The second objection is that prospective parents wishing to access MRTs could say that they intend to disclose and then once the child is born they could simply break off contact with the clinic and choose to never disclose to the child. This is a difficult challenge that any clinic might face and the UK Department of Health has made clear that “the regulation-making power does not provide the scope to include this (i.e. a requirement for follow-up research) within the regulations and, in any case, there would be difficulties around placing a legal obligation on families to participate in follow-up research” (2014a: p. 24) or any clinical monitoring for that matter. However, offering counselling prior to using MRTs may also help parents to better understand the risks of non-disclosure to their children in this instance, as well as the potential health risks of breaking off contact with medical experts. In any event, having a small number of parents changing their minds after the use of MRTs and ultimately not disclosing is preferable to making disclosure mandatory. This is true not only because it is difficult to enforce mandatory disclosure from a practical point of view (as mentioned above) but also because it would be challenging to legally justify such interference in the private and family life of the families created via MRT use.

For example, enforcing a mandatory disclosure policy in the case of MRT use would likely contravene Article 8 of the European Convention of Human Rights (ECHR), which states:

1. Everyone has the right to respect for his private and family life, his home and his correspondence.
2. There shall be no interference by a public authority with the exercise of this right except such as is in accordance with the law and is necessary in a democratic society in the interests of national security, public safety or the economic wellbeing of the country, for the prevention of disorder or crime, for the protection of health or morals, or for the protection of the rights and freedoms of others.

The question of whether an interference is justifiable under Article 8(2) involves a three-step analysis which addresses whether the measure is in accordance with law, has a legitimate aim, and is necessary.^22^ In turn, the third element involves a three-step assessment, established in *Sunday Times* v *United Kingdom*,^23^ namely whether there is a pressing social need for the interference, whether it is proportionate to the legitimate aim and whether there are relevant and sufficient reasons for it. At this point in time, given the stringency of the test to justify interference under Article 8(2), justifying compulsory legal disclosure would require more robust scientific evidence about the possible risks associated with being MRT-conceived than is currently available.

### Sex selection to reduce health risks to future generations

There is uncertainty about the safety of MRTs for future generations, and an ethical responsibility therefore exists to employ a safety strategy in clinical practice, which would help reduce transgenerational health risks. One concern is that during PNT or MST, small amounts of mitochondria with harmful mtDNA mutations could accidentally be transferred, along with the maternal spindle or pronuclei, and deposited into the healthy egg or embryo, respectively (Bredenoord et al. 2010: p. 1355; NCB 2012: p. 80). Having such a small amount of diseased mitochondria would be unlikely to cause any suffering to the person created via the MRT. However, if a person carrying very low levels of unhealthy mtDNA has children, there is a possibility that a mtDNA disease could manifest in the those offspring. Because the mitochondrial genome is maternally inherited, clinicians applying to the HFEA for a license to use MRTs should be required to use sex selection techniques to select for male offspring (whenever possible) (Bredenoord et al. 2010)^24^ in order to reduce mtDNA-related transgenerational health risks.^25^

Traditionally, PGD has been the method by which sex selection has been carried out on embryos. However, in 2013 the HFEA advised the UK government that sex selection via PGD is an impractical safeguard because it requires additional manipulation of the embryo (HFEA 2013a: p. 17). If PGD for sex selection is not an option, then clinicians could use sperm sorting technology instead. Sperm sorting using ‘flow cytometry’ is a process in which a fluorescent chemical binds with the DNA of sperm cells and a laser is then used to sort ‘female’ sperm cells with X-chromosomes from ‘male’ sperm cells with Y-chromosomes. A laser is able to detect and sort the two cell types because X-chromosome bearing cells appear more fluorescent than Y-chromosome bearing sperm cells, because the former have more DNA for the fluorescent chemical to bind to. However, this type of sperm sorting can only be used by clinics which are licensed by the HFEA to use it with patients for medical reasons.

The main drawback of sperm sorting is that it is not 100 % reliable. One sperm sorting technology is MicroSort^®^ and it will correctly sort sperm into samples that are on average 91 % pure ‘female’ sperm and 74 % pure ‘male’ sperm (Microsort 2014). It is possible that these figures might improve as sperm sorting technologies are refined in the coming years. However, even if clinicians used sperm sorting to successfully create male embryos only 74 % of the time, this would still represent a potential 24 % reduction of mitochondria related transgenerational health risks in a clinical trial (assuming that the average ‘natural’ likelihood of having a female or male child is about 50 %). Clinicians should therefore use sperm sorting for sex selection to create the first generation of persons with MRTs, as this would help to reduce risks to future generations.

However, in a report on MRTs published by the NCB, Ken Taylor and Erica Haimes, responding to the Nuffield Working Group’s call for evidence, identified several objections to the prospect of using sex selection for the first clinical use of MRTs (NCB 2012: p. 80). First, they argue that it would be unacceptable to create an ‘experimental group’ of male offspring who would have to be monitored over the course of their lives (NCB 2012: p. 80). Taylor and Haimes express concern that as a result of sex selection there would be a generation of boys conceived via MRTs who would be ‘experiments’ and would “…live with uncertainty about their future health, beyond that normally experienced” (NCB 2012: p. 80). They add that these individuals would need to be considered healthy before the next generation of children (including females), could be created with MRTs (NCB 2012: p. 80). Second, they argue that the selection of only males would be based on the assumption that the long-term undesirable outcomes associated with MRTs would not be attributable to anything beyond mitochondria (NCB 2012: p. 80). On the basis that this particular assumption could be wrong, they argue that researchers cannot justify only exposing male offspring to the potential risks of MRTs (NCB 2012: p. 80). Finally, they suggest that the mere fact that sex selection is being considered as a means of limiting risk, is itself indicative that not enough is yet known about whether MRTs are safe (NCB 2012: p. 80). The above are all important points worth considering.

Consider the objection that it would be unethical to create an experimental group of male offspring who would be studied for a large part of their lives. As noted earlier, it would be valuable to monitor MRT-conceived persons for as long as possible in order to be able to record any adverse results that might emerge about MRTs (DH 2014b). But while parents who have children via MRTs may feel morally compelled to have their children monitored by clinicians, doing so would ultimately be their own choice because compulsion of this obligation would be difficult for a range of legal, policy and practical reasons (DH 2014b). That said, any voluntary clinical monitoring program for MRT-conceived persons would be of value to parents wishing to care for the wellbeing of their children. In addition, it is unclear how demanding the role of being monitored by clinicians might actually be for children or adults. It might be that being clinically monitored is not very demanding. Nevertheless, at the age of 18 anyone who was initially enrolled in such a monitoring program because they were conceived via an MRT could opt out if they wished. Before the age of 18, they may be able to do so regardless of their parents’ desires for them to remain involved, depending on how serious the risks of non-participation were deemed to be at the time.^26^ MRT-conceived persons could also suffer from the uncertainty of not knowing if they will have future health complications as a result of having been MRT-conceived. While all of the above concerns are ethically relevant for researchers to keep in mind, none of these concerns are specific to the use of sex selection. In fact, none of these concerns would be resolved if sex selection were not used and if the children conceived via MRTs were of both sexes. The demandingness of being born into a life which involves clinical monitoring (or any form of follow-up programme) is unaffected by whether or not the offspring are solely male, and as a result this argument is not a good reason for not recommending the use of sex selection as a licensing condition for the first use of MRTs in clinics.

The second objection to sex selection claims that because researchers are not yet certain that mitochondria will be the root cause of any possible undesirable side effects following the use of MRTs, we therefore cannot justify only selecting for male children to be born in order to safeguard against this ‘assumed’ risk. This is an interesting argument, but it neglects several other important points that make sex selection an important research requirement. As noted above, evidence to date has indicated that there could be a genuine risk that during the procedure of using an MRT, diseased mitochondria could be unintentionally transferred into a healthy egg or embryo. If the evidence continues to suggest that there could be a mitochondria-linked health risk to future generations, then sex selection would remain an important safeguard for clinicians to employ.

The third objection claims that if sex selection is a necessary safety precaution for clinical MRT use at this point in time, then this is indicative that these techniques should not be used because not enough is known about their safety. However, even if the recommendation of sex selection does trigger concerns from critics about the safety of MRTs, the other way of looking at this is that the absence of this recommendation should also trigger concerns about the lack of an important safeguard for the wellbeing of future generations. It would be a serious mistake to make broad assumptions about the overall safety of the clinical use of MRTs on the basis that sex selection also happens to be a viable method of safeguarding future generations from suffering mitochondria-linked disorders. Any appraisals of the safety of MRT use should instead be based on a full review of the evidence, rather than speculation that is premised on the proposal of an individual clinical intervention such as sex selection.

Considering how few prospective parents would likely be granted clinical access to MRTs (the chances are that it would be <10 cases each year), it is hard to imagine how bringing only males into existence would harm society or cause suffering to the prospective parents or children (DH 2014a: p. 38). The small number of licenses granted by the HFEA for MRT use would mean that the use of these techniques would not have an impact on sex ratios in the general population, especially considering that the rarity of mtDNA disorders may entail the recruitment of individuals from a broad geographic area. As sex selection would only be used for the sake of safety, it could hardly be construed as an expression of sexism. Creating a cohort of MRT-conceived children who are all male would of course also mean that only evidence on the welfare of males could potentially be gathered by any form of future social science research or clinical monitoring conducted on this cohort. Even so, this would be a worthwhile limitation if it further demonstrates that the techniques are generally safe and if it provides additional confidence that MRTs will most likely be safe to use for both the creation of females and any generations of future offspring they may have.

Some countries, such as the UK, do not permit sex selection for the sake of avoiding the creation of carriers who will not suffer from inheritable diseases but may pass them on to their offspring. Under Schedule 2 of the Human Fertilisation and Embryology (HFE) Act (1990) (as amended by the HFE Act 2008):

- 1   (1) A licence under this paragraph may authorise any of the following in the course of providing treatment services—
  …
  - (b)   procuring, keeping, testing, processing or distributing embryos…
  1ZA (1) A licence under paragraph 1 cannot authorise the testing of an embryo, except for one or more of the following purposes—
  …
  - (c)   in a case where there is a particular risk that any resulting child will have or develop—
    (i) a gender-related serious physical or mental disability,
    (ii) a gender-related serious illness, or
    (iii) any other gender-related serious medical condition, establishing the sex of the embryo…
- (2)   A licence under paragraph 1 cannot authorise the testing of embryos for the purpose mentioned in sub-paragraph (1)(b) unless the Authority is satisfied—
  - in relation to the abnormality of which there is a particular risk, and
  - in relation to any other abnormality for which testing is to be authorised under sub-paragraph (1)(b),
  that there is a significant risk that a person with the abnormality will have or develop a serious physical or mental disability, a serious illness or any other serious medical condition.
- (3)   For the purposes of sub-paragraph (1)(c), a physical or mental disability, illness or other medical condition is gender-related if the Authority is satisfied that—
  - it affects only one sex, or
  - it affects one sex significantly more than the other.

What constitutes a significant risk in the realm of mtDNA disease can be difficult to determine. According to the HFEA Code of Practice, decisions about what constitutes ‘significant risk’ are left up to the judgement of clinicians in the case of PGD (HFEA 2014: §10.6). Furthermore, the Department of Health (2014a: p. 46) has indicated that any future use of MRTs on humans is likely to be approved for licensing on a case-by-case basis.

Since, as noted earlier, PGD for sex selection is not an option, we must consider the regulation of sperm sorting. As a matter of interpretation of the HFE Act (1990) (as amended), sex selection via sperm sorting must be licensed by the HFEA before it can be used by a clinic (HFE Act 1990: Schedule 2, ¶ 1ZB(3)).^27^ Given the terms of the current sex selection regulations regarding embryos just discussed, the HFEA would be unlikely to consider a low level mtDNA mutation carrier to be at a ‘particular’ or ‘significant’ risk of developing a ‘serious physical or mental disability, a serious illness or any other serious medical condition’ (as required under the HFE Act 1990: Schedule 2, ¶ 1ZA(2)). Nevertheless, the use of sex selection using sperm sorting in conjunction with MRTs could be made legally permissible if the recently approved HFE Regulations 2015 for MRTs were amended (and the amendments were then passed into law by Parliament). In the HFE Regulations 2015 (which amend the HFE Act 1990, as amended) an amendment could be made in Part 2, Section 9, titled “Supplemental provision-licenses”, to include an additional provision that would allow for the use of sex selection to avoid or reduce the transmission of mtDNA diseases in the context of MRT use on humans. The amendment of the regulations may appear to be a demanding recommendation. However, permitting sex selection would assist clinicians to fulfil their ethical duty to prospective parents and their future offspring, by minimising the possible transgenerational health risks of using MRTs. Regulators also have a responsibility to enable clinicians to conduct safe clinical procedures and in this instance that means permitting the licensed use of sperm sorting for sex selection.

## Conclusion

The aim of this paper has been to clarify and critically assess some of the central ethical concerns about the safety of MRTs for clinical use and to then move the debate forward by focusing on two key recommended conditions that should be met in order for the HFEA to grant clinicians a license to use MRTs on humans. These recommended conditions will help to reduce the potential health risks of MRTs. The future human use of MRTs has been dismissed by some as ethically impermissible because other safe alternatives, such as egg donation, PGD and PND, already exist. However, for some intending parents who want offspring to whom they are genetically related, but who face the prospect of passing on a mtDNA disease, no other reproductive alternatives exist beyond the prospect of using MRTs.

Two types of safety concerns about offspring having three genetic ‘parents’—or more accurately, three genetic contributors—regularly emerge in discussions about the clinical use of MRTs. The first concern is that MRT-conceived offspring could experience some form of psychosocial suffering, but this concern is not persuasive because existing empirical evidence suggests that persons in similar situations (e.g. gamete donor conceived persons) usually experience average psychosocial wellbeing (Freeman 2015; NCB 2013). The second concern is that the physical wellbeing of MRT-conceived persons could suffer as a result of mitochondria-related health complications (e.g. mtDNA that is incompatible with nuclear DNA or low levels of mitochondria with diseased mtDNA). Concerns regarding health risks to future MRT-conceived persons should be taken seriously and action should be taken by researchers, regulators and clinicians to reduce such risks in at least two ways: (1) adequate pre-clinical safety testing of MRTs needs to be carried out; and (2) the framework for licensing the first clinical use of MRTs should be well-constructed with the aim of reducing or eliminating health risks wherever possible. While the HFEA has a schedule of pre-clinical research milestones in place to test the safety of MRTs, comparatively little progress has been made in terms of discussing how the licensing conditions and framework for the first clinical use of MRTs should be designed in order to reduce MRT-related health risks.

This paper recommends two licensing conditions that should become part of any future licensing framework used by the HFEA to permit the clinical use of MRTs with humans. The first recommended licensing condition is that any child conceived following the use of a MRT should be disclosed to by adulthood, either by their parents or by a member of the clinic (e.g. clinician or counsellor). In fact, prospective parents who do not intend to disclose, should be excluded from accessing MRTs in clinics. One advantage of this recommendation is that the MRT-conceived children’s autonomy will be improved as a result of knowing a key piece of their medical history, which they can use to help care for themselves or to inform others (e.g. physicians) to help care for them if a MRT-related health complication arises. In addition, MRT-conceived persons will be better informed about the potential health risks of their own reproductive decisions (e.g. will their children inherit a mtDNA disease?). A second advantage is that if children are disclosed to they will then be aware of the nature of any possible research their parents may have become involved in and at the age of 18 researchers will be in a better position to talk with them about continuing with a programme of medical monitoring or social science research. Disclosure better enables the long-term medical monitoring of MRT-conceived offspring, and both those being monitored and future generations will benefit from any findings discovered in this initial monitoring or research.^28^ This recommendation for disclosure should be incorporated into the guidance of the HFEA Code of Practice (2014) and it should be included in any additional guidance documents on the clinical use of MRTs produced by either the HFEA or the UK Department of Health.

The second recommended licensing condition is that MRTs should only be permitted for clinical use if clinicians agree to use sex selection, with the aim of only creating male embryos in order to reduce or eliminate the likelihood of transgenerational mitochondria-related health risks. Objections exist to this recommendation; however, none of them outweigh the risks that are reduced by sex selection. It is unlikely that sex selection would be licensed for MRT research under the current law (i.e. Schedule 2 of the HFE Act 1990, as amended 2008), and therefore the HFE Regulations 2015 should be amended to include a provision that permits the use of sex selection in conjunction with the clinical use of MRTs. This recommendation has the advantage of reducing the likelihood that future generations will suffer mitochondrial-related health complications and it also saves MRT-conceived female offspring the future reproductive concern of potentially passing on inheritable diseases to their own offspring (Bredenoord et al. 2010: p. 1354).

This paper responds to the need for further ethical discussion about how regulators should improve the safety and minimise the risk of making MRTs clinically accessible for human use for the first time. Importantly, this paper also calls for further discussion on both the ethical issues it raises and the recommendations it offers. The process of developing a robust framework for licensing the clinical use of MRTs will be challenging and contentious, and this process should be afforded ongoing scientific and ethical scrutiny.

The recommendations made in this paper are discussed in the context of UK regulations for MRTs and in relation to how the techniques should be licensed by the HFEA for use in UK clinics; however, these recommendations are also relevant to international contexts (e.g. US or Sweden). As MRTs and new novel ARTs like them are increasingly developed and made clinically available, it is important that countries, such as the UK, demonstrate the willingness and capacity for detailed ethical review while these techniques make their way from bench to beside. The perceived success of the HFEA’s ethical and scientific review of MRTs (i.e. licensed clinical use) may impact broader societal attitudes towards the moral permissibility of introducing future ARTs. The ethical debates and recommendations discussed in this paper are an important part of setting a precedent of rigorous discussion which will inevitably inform expectations for debate surrounding the introduction of future technologies.^29^

As researchers approach the first use of MRTs on humans, more work remains to be done in order to ensure that the licensing framework in place for clinics is ethical by virtue of it minimising risks whenever possible. Taking seriously the arguments and recommendations put forward in this paper is an important step towards meeting this ethical challenge.

## Footnotes

1 Conception occurs after MST and before PNT; however, in this paper I refer to anyone brought into existence with the help of MST or PNT as being ‘MRT-conceived’.

2 The UK Department of Health (2014b: p. 42) has emphasised the role and significance of follow-up monitoring and research for MRT-conceived families, as part of its concluding consultation report on draft regulations.

3 As discussed later, the wording of ‘increase the likelihood’ is used here instead of ‘guarantee’, because sperm sorting is what is recommended and it is not 100 % reliable.

4 An asymptomatic female carrier of a homoplasmic mtDNA mutation may subsequently pass on the mtDNA mutation with the prospect of it causing her offspring considerable harm.

5 In basic terms the ‘mutation load’ refers to the ratio of particular mutant to non-mutant genes that exist in a person’s body. For example, an mtDNA mutation load of 90 % for mutation ‘X’ would mean that mutation ‘X’ is present in 90 % of the mtDNA in a body.

6 In the context of heteroplasmic mtDNA disorders, a genetic bottleneck is created when mitochondria divide during cell division and each new mitochondrion takes some of the original mitochondrion’s mtDNA with it. After division both the old and new mitochondria replicate the copies of mtDNA they were left with after division. As a result, there are varying levels of mutant mtDNA across the mitochondria in our bodies because different ratios of mutant to healthy mtDNA are passed on each time a mitochondrion divides. The term ‘mosaicism’ refers to the different levels of mutant mtDNA that exist between different mitochondria, and ultimately the different cells that carry them.

7 During PNT it remains unclear if (and how often) a small quantity of the mitochondria with mutant mtDNA are transferred from the discarded embryo and into the new embryo.

8 PDD is a spectrum disorder and refers to a group of autism-related disorders (NCB 2012). Turner Syndrome affects females and can involve a number of symptoms, such as: lymphedema (swelling), sterility, and hearing loss.

9 This point has also been discussed in detail by the NCB (2012: pp. 57–87) and Bredenoord and Braude (2011).

10 MRT-conceived offspring would have ties through their nuclear genome to two persons and a tie through their mitochondrial genome to a third person.

11 The term psychosocial refers to persons’ psychological and behavioural development in relation to their social environment.

12 The UK Department of Health (2014b) has emphasised the importance of carrying out similar research in the future on the first generation of MRT-conceived persons.

13 In this debate there has been considerable disagreement about whether or not MRTs should be considered a form of germ-line modification or a form of genetic modification. The UK Department of Health (2014a) and the NCB (2012) argue that because MRTs only introduce new mtDNA and do not modify the nuclear genome, it is incorrect to describe MRTs as a form of genetic modification and that it is in fact a form of germ-line modification. Meanwhile, Baylis (2013: p. 533) and Knoepfler (2014) interpret the replacement of the intending mothers’ mitochondria (and the genome they carry) with a donor’s mitochondria, to be a form of genetic modification. In the end, if the potential health outcomes of using MRTs is our only concern, then the question of whether or not PNT or MST are forms of germ-line modification or genetic modification, is actually a moot point because alone this distinction will not reveal anything about whether or not the science is safe.

14 This point was discussed and debated at length in a UK House of Commons Science and Technology Committee (2014) meeting on evidence about mitochondrial donation. It was also raised in an article by Reinhardt et al. (2013) and responded to by the HFEA (2013b) in a statement which offers a technical discussion of the safety concerns. Ted Morrow has also been concerned about the safety of possible mismatches between nuclear DNA and mtDNA and that MRT regulations and research to date have not adequately accounted for this concern (Morrow 2014b). The HFEA agrees that mtDNA and nuclear DNA interactions are still poorly understood and risks may exist as a result of mismatches (and that perhaps some form of attempt at matching DNA types is needed in the future, when possible); however, it is not clear that the risks associated with mismatches between the mtDNA and the nuclear DNA are going to be greater than any other form of human reproduction and it is not clear that MRTs will carry any heightened risks (in this respect) (HFEA 2013b).

15 For further discussion see Braude and Lovell-Badge (2014) in conversation with Knoepfler (2014).

16 Here the act of telling someone that they were MRT-conceived is referred to as ‘disclosure’.

17 If MRT-conceived children are not disclosed to, it is possible that they could suffer from the stress and anxiety of thinking they will inherit a debilitating mtDNA disorder from their mothers. A recent NCB report (2012) highlighted a similar case in the context of sperm donor conception, where a child grew up with the stress and anxiety of thinking she might fall ill from an inheritable disorder from her father. The child was not genetically related to the social father; however, because of non-disclosure by her parents she suffered the psychological harm that came with the false belief that she may have inherited a debilitating condition.

18 There are several reasons for this. The first is that few clinics are likely to be licensed to use MRTs. The second is that few clinicians will initially have adequate training to use MRTs.

19 For example, their families may have simply told them they were being researched for a reason other than having been conceived via an MRT.

20 Some parents may choose to disclose to their children from a very young age (and continue to discuss this with them from that time onward) so that there is never a time when their children were not told about being MRT-conceived (in some sense); however, other parents may wait until the ‘right time’ (e.g. early adolescence) to disclose to their children because the parents feel that they should wait to disclose at a stage of their children’s lives when they are more capable of grasping the meaning of being donor-conceived. For a discussion on the complexities of disclosure, see: NCB (2013).

21 Clinics are always likely to screen gametes for inherited genetic disorders (e.g. Huntington’s disease) regardless of whether or not MRTs are likely to be used. Therefore, the threat of future persons inheriting genetic disorders as a result of IVF in a clinic is always likely to be extremely low.

22 See e.g. *A, B & C* v *Ireland*, App No 25579/05, (2011) 53 E.H.R.R. 13 paras 219–242.

23 *Sunday Times* v *United Kingdom* (1979) 2 E.H.R.R. 245.

24 Sex selection technology such as sperm sorting is not 100 % reliable.

25 Bredenoord et al. (2010) were some of the first to give a detailed account of the merits and weaknesses of using sex selection (including sperm sorting) to manage risks in conjunction with the use of MRTs.

26 Adolescents are presumed competent by virtue of the Mental Capacity Act (2005), s. 1(2). The Act applies to anyone over 16 (s. 2(5)). However, case law shows that the courts are reluctant to let adolescents refuse medical treatment where this may result in serious harm to them. See especially *Re W**(A Minor) (Medical Treatment: Court’s Jurisdiction)* [1993] Fam 64 and the judgments of Balcombe and Nolan L.J.J. That case concerned, in part, interpretation of the Family Law Reform Act (1987). 8(3): the court held that doctors can rely on a parent’s consent in the face of a refusal by an adolescent of 16/17 years (where that would result in serious harm to them). When a person reaches the age of 18, the courts no longer intervene in this way. In addition, note that the legal presumption of a lack of competence under 16 is rebuttable by virtue of the decision in *Gillick* v *West Norfolk & Wisbich Area Health Authority* [1985] UKJL 7.

27 Section 10.19 of the HFEA Code of Practice (2014) also states “If sperm is sorted for medical reasons to create (or maximise the chance of creating) embryos of a particular sex for medical reasons, patients should be given information about the process, procedures, possible risks and the experience of the clinic in doing the procedure.” This is helpful because it sheds light on the best clinical practices for sperm sorting and it also suggests that Schedule 2, paragraph 1ZB(3) of the HFE Act (1990) (as amended) is indeed referring to the licensing of sperm sorting as one of the “other practices designed to secure that any resulting child will be of one sex rather than the other…”.

28 Unfortunately, no follow-up research was conducted on children conceived with CT and as a consequence there is no published data available on the development and well being of this cohort.

29 For a discussion of additional ethical concerns related to MRTs (but beyond the scope of this paper), see: NCB (2012). Other examples of ethical concerns include: MRTs amounting to eugenics and being incompatible with human dignity (see Appleby et al. 2013; Brice 2013); MRTs involving the destruction of human embryos in research and treatment (see Caldwell 2015); and MRTs leading to a slippery slope towards other more undesirable treatments being permitted (see Wong and Johnson 2014). However, these concerns are also beyond the scope of this paper.
